# Oxidative stress parameters as biomarkers of bladder cancer development and progression

**DOI:** 10.1038/s41598-021-94729-w

**Published:** 2021-07-23

**Authors:** Paulina Wigner, Beata Szymańska, Michał Bijak, Ewa Sawicka, Paweł Kowal, Zofia Marchewka, Joanna Saluk-Bijak

**Affiliations:** 1 Department of General Biochemistry, Faculty of Biology and Environmental Protection, University of Lodz, Pomorska 141/143, 90-236 Lodz, Poland; 2 Department of Toxicology, Faculty of Pharmacy and Division of Laboratory, Wroclaw Medical University, Borowska 211, 50-556 Wrocław, Poland; 3 Biohazard Prevention Centre, Faculty of Biology and Environmental Protection, University of Lodz, 90-136 Lodz, Poland; 4 Department and Clinic of Urology and Urological Oncology, Faculty of Postgraduate Medical Training, Wroclaw Medical University, Kamieńskiego 73a, 51-124 Wrocław, Poland

**Keywords:** Biochemistry, Biomarkers, Oncology, Urology

## Abstract

The epidemiological studies confirm that the overproduction of free radical is an important factor of cancer induction as well as development, and loss of antioxidant systems efficiency is associated with an increased risk of carcinogenesis. While bladder cancer is the fourth most common type of cancer all over the world, there is little evidence of the advancing changes in oxidative/nitrative stress during the progression of bladder cancer. Our study aimed to investigate the plasma levels of typical markers of oxidative/nitrative stress depending on the clinical classification of bladder cancer differentiation and infiltration degree. We examined 40 patients with newly diagnosed bladder cancer and 20 healthy volunteers as a control group. We analysed the plasma levels of protein carbonyls, thiol groups, 3-nitrotyrosine, lipid peroxidation, as well as non-enzymatic plasma antioxidant capacity using DPPH^**·**^ and ABTS^**·**+^ radicals. We confirmed that all analysed biomarkers are higher in enrolled BC patients than in healthy subjects. Furthermore, our findings demonstrate a positive correlation between the degree of bladder cancer progression and the level of oxidative stress, but no correlation in the case of NT-3. Based on obtained results, we might conclude that during carcinogenesis of the bladder increased oxidative damage of biomolecules is manifested. This indicates the participation of oxidative stress in the development of bladder cancer, and it is important the ensure the proper antioxidant protection.

## Introduction

Bladder cancer (BC) is one of the most commonly diagnosed cancers among people aged over 65 years. BC is the fourth most common type of cancer in men and the eighth-most commonly diagnosed malignancy in women worldwide^1^. Unfortunately, the number of new cases continues to increase, especially in developed countries. In the Western World, the BC is the 4th most common cancer in men and the ninth in women. It was estimated that 550,000 people were diagnosed with BC in 2018. Moreover, nearly 200,000 people died of BC in 2008. Interestingly, men are four times more likely than women to be diagnosed with BC^2–5^. Nevertheless, information about the BC pathogenesis is still incomplete, but some scientific reports indicate the ROS involvement in the formation, development and progression of this cancer. In previous studies, it has been demonstrated that reactive oxygen/nitrogen species (ROS/RNS) have been involved in the development of various kind of cancer due to irreversible damage of all types of cellular and extracellular macromolecules, including DNA damage lead to mutations^6,7^. Furthermore, ROS/RNS are identified as molecules especially involved in not only carcinogenesis but also metastasis processes. A high level of ROS/RNS can stimulate cancer cells to form metastatic colonies. The cytotoxic nature of ROS/RNF is responsible for affecting the invasion and adhesion processes of cancer cells. The involvement of ROS/RFS in the signalling pathways leads to upregulation of gene expression, including genes responsible for the metastasis and proliferation of cancer cells. ROS/RNS overproduction determines the maintenance of the inflammatory microenvironment favourable to carcinogenesis^7–10^. It is well known that the injurious effects of ROS/RNS are controlled by various defence systems consisting of enzymatic and non-enzymatic mechanisms. The epidemiological studies reveal that the depletion in the activities of antioxidants is associated with an increased risk of carcinogenesis^11^. However, it is hard to indicate whether the ROS overproduction induces a cancer transformation or whether it is a consequence of it. In the case of bladder cancer, exposure to environmental and occupational chemicals may induce an increased production of ROS. The high ROS level may stimuli the production of proinflammatory cytokines and proangiogenic factors, including TNF-α, VEGF and MMP-9. On the other hand, bladder cancer development may be the result of prolonged inflammation. The inflammation microenvironment showed the large concentration of ROS and RNS releasing by activated macrophages and lymphocytes. Consequently, a high ROS and RNS concentration may cause DNA mutations of proliferating cells, including bladder epithelial cells^12,13^. These mutations may be localised in oncogenes and suppressor genes involved in carcinogenesis, such as *Ras* and *p53*. The obtained results confirmed that *Ras* mutation was identified in about 30% of BC cases, whereas the *p53* mutation was observed in over 50% of BC patients^14,15^. Moreover, both oxidative stress and inflammation may stimuli cancer progression by angiogenesis induction. Angiogenesis is the crucial development process of new blood vessels from pre-existing vessels, which allows for tumour progression and metastasis. Previous studies confirmed that ROS might modulate the expression of proangiogenic factor, including VEGF and MMP-9, by increased binding of transcriptional factors to its promoter regions^16^. Similarly, TNF-α as a proinflammatory cytokine impacted the transcription of MMP-9 by stimulated its 5′-flanking promoter activation^17^. Moreover, cytokines may impact angiogenesis by the inhibitions of apoptosis. TNF-induced NF-κB activation causes the transcription of antiapoptotic factors, including A20, cIAP-1, cIAP-2, Bcl-xL, XIAP and IEX-1L^18^. Similarly, IL-6 by activated protein kinase B (AKT) may lead to an increase in cell survival and an inhibition of apoptosis^19^. Therefore, oxidative stress involvement in BC development is not negotiable, but the precise role is still unclear. Nevertheless, blood biomarkers of oxidative stress may be a potential biomarker of diagnosis and prognosis of BC^7,20,21^.

Thus, our attention has been focused on the association between the level of oxidative/nitrative stress and the clinical classification of cancer differentiation and infiltration degree. This study aimed to investigate the oxidative/nitrative stress status by determining plasma levels of protein carbonyls, thiol groups and 3-nitrotyrosine (3-NT)—as markers of protein oxidative/nitrative damages, TBARS level as a marker of lipid peroxidation, as well as non-enzymatic antioxidant capacity of blood plasma using DPPH^**·**^ and ABTS^**·**+^ radicals, to explore the role of oxidative/nitrative stress in the BC development and progression. We have focused on establishing the range of oxidative/nitrative stress biomarkers in the two groups of patients that differ in the progression of bladder cancer: TaG1 (well-differentiated not invasive tumour papillary) and T1G2 (medium differentiated tumour infiltrating the mucosa). Moreover, based on the previous reports, we undertook a multi-parameter analysis, which allows identification of potential the most sensitive marker of BC development.

## Materials and methods

### Patients and volunteers

Forty patients with newly diagnosed bladder carcinoma admitted to the Department and Clinic of Urology and Urological Oncology, Wroclaw Medical University and twenty healthy volunteers were investigated. The mean age of women with BC was 69, and the mean age of men with BC was 70. In the case of the control group, the mean age of women was 66, while the mean age of men was 69. The characteristics of the patients had presented in Table 1.

**Table 1:** Demographic and clinical characteristics of patients and control subjects.

Characters	Control		BC patients		p value
	No	%	No	%	
**Age (years)**					
40–60	6	30	6	15%	p = 0.2040
> 60	14	70	34	85%	
**Gender**					
Females	4	20	8	20%	p = 0.3508
Males	16	8	32	80%	
**T stage and G grade**					
TaG1	N/A		16	40	N/A
TaG2			4	10	
T1G2			14	35	
T2G2			4	10	
T2G3			2	5	

The p-values were calculated using the Student’s t-test.

The control group was composed of healthy volunteers with no history of cancer or chronic inflammation. The BC patients and subjects from the control group were of similar socioeconomic status. Additionally, they were people who have never been diagnosed with cancer or other chronic disease and without any chronic inflammatory. Based on a questionnaire conducted among patients, the majority of them were smokers (67%). Similarly, in the control group, smoking accounted for 63%. Moreover, 70.6% among patients are the urban dwellers and 18.2% reported the contact with pesticides. Exposure related to the occupation or the place of residence in the industrial area was declared by 41.2%. The questionnaire showed that patients had different dietary preferences. The patients were asked a question about the consumption of smoked and grilled products, the predominance of meat dishes in the diet or the amount of vegetables consumed. 45% of patients declared a large amount of meat dishes consumption, 25% smoked and grilled dishes, while the advantage of a vegetable diet over meat only 12% of patients.

The volunteers taking part in the experiment were native Poles from south Poland. Due to the clinical stage, patients were classified according to the scale TNM (Tumor, Node, Metastasis). The histological grade of the tumour (histological grade of malignancy) was defined according to WHO grading from 1973. On this basis, the patients were divided into subgroups. In forty patients, TaG1 tumour was observed in sixteen people, TaG2 tumour in four, T1G2 tumour in fourteen participants and T2G2 tumour in four, whereas two cases had T2G3 tumour.

All participants took part in the study voluntarily and provided written informed consent before participation. All procedures had done according to the Helsinki Declaration and approved by the Ethics Committee of the Medical University of Wroclaw Medical University, Poland KB-12/2014.

### Blood samples collection

All whole blood samples had collected in the morning (between 7.00 a.m. to 9.00 a.m.) in fasting status and stored using the same protocol. The venous blood samples (3 mL) were collected from an ulnar vein into 3.2% buffered sodium citrate containing tubes (BD Vacutainer, Becton, Dickinson and Company, Franklin Lakes, NJ, USA) and immediately centrifuged to isolate plasma (15 min, 2000 × *g*) at room temperature in accordance with the manufacturer's tube instruction. Then, the plasma prepared in this way was used in subsequent analyses or stored at − 80 °C.

### Measurement of carbonyl groups by ELISA method

The presence of carbonyl groups indicates protein peroxidation. Detection of carbonyl groups by ELISA method was performed in plasma and estimated as adducts of 2,4-dinitrophenylhydrazine (DNPH), according to the method described by Buss et al*.* and modified by Alamdari et al.^22,23^. Firstly, the microplates were incubated overnight at 4 °C to non-specifically adsorption of plasma proteins onto ELISA plates. Secondly, the wells had washed with 300 µL PBS, and then human plasma proteins reacted with substrate 2,4-dinitrotrophenylhydrazine (DNPH; 0.05 mM, 200 µL, pH 6.2). The plate was incubated at room temperature in the dark for 45 min and was washed 5 times with 300 μL PBS: ethanol (1:1, v/v) and last time with 300 μL PBS. The carbonyl groups had detected by the anti-DNP antibodies and then by the second antibodies conjugated with horseradish peroxidase. This sensitive method was calibrated using oxidized albumin and required only 5 μg protein per well. The oxidized albumin had used to prepare the standard curve. The construction of a standard curve expressed as nmol of carbonyl groups/mg of albumin had used to confirm the linearity of the ELISA method. The level of carbonyl groups was determined by spectrophotometrically (λ = 316 nm) according to Levine et al.^24^. Finally, the carbonyl groups level was expressed as mg/mL of plasma proteins. The concentration of plasma proteins (mg/mL) was determined using the BCA method, according to the method described by Walker et al.^25^.

### Determination of thiol groups by Ellman method

Under physiological conditions, thiol residues in the reduced state, while ROS and RNS could lead to the formation of disulphides. The level of thiol groups indicates the intensification of the process of oxidative stress^26^. The total sulfhydryl groups in plasma had measured by the method original describing by Ellman and modified by Hu^27,28^. In this method, thiols interact with the 5,5′dithiobis-(2-nitrobenzonic acid) (Ellman’s reagent, DTNB) and the highly coloured anion 2-nitro-5-thiobenzoate (TNB) is formed. TNB^-^ ionizes to the TNB^2−^ di-anion, which has a yellow colour. TNB^2−^ is quantified by measuring after 1-h incubation in 37 °C the absorbance at 412 nm. The concentration had calculated by using the molar extinction coefficient for thio-nitro-benzoic anion (TNB) (ε = 13,600/M/cm). The sulfhydryl groups concentration was expressed as mM.

### Detection of 3-nitrotyrosine by competitive ELISA method

3-Nitrotyrosine is formed as a product of tyrosine nitration mediated by reactive nitrogen species, including peroxynitrite anion and nitrogen dioxide^29^. Detection of 3-NT in the plasma proteins was performed according to the Khan et al. by the competitive ELISA test^30^. Then, the 3-NT amount was expressed as mg/mL of plasma proteins. The concentration of plasma proteins (mg/mL) was determined using the BCA method, according to the method described by Walker et al.^25^. Wells of a 96-well microtiter plate was coated with nitro-fibrinogen (nitro-Fg; concentration 1 µg/mL and 10 mol of 3-NT/mol of protein) overnight at 4 °C and has been blocked with skim milk to prevent non-specific binding. Based on the standard curve drawn up of the 3-nitrotyrosine containing fibrinogen (3-NT-Fg), the concentrations of nitrated plasma proteins had assessed. To receive the 3-NT-Fg, the human fibrinogen had treated with the peroxynitrite at the final concentration of 1 mM. The amount of 3-NT in fibrinogen was determined spectrophotometrically (λ = 430 nm; ε = 4.400/M/cm). After the spectrophotometric measurement obtained nitro-fibrinogen was used to prepare the standard curve, ranging from 10 to 1000 nM/L of 3-nitrotyrosine-fibrinogen equivalent.

### Evaluation of lipid peroxidation level

Plasma samples were mixed with an equal volume of 15% (w/v) cold trichloroacetic acid in 0.25 M HCl and with an equal volume of 0.37% (w/v) thiobarbituric acid (TBA) in 0.25 M HCl. Then, samples were immersed in a boiling water bath for 10 min, and after cooling, samples were centrifuged and then the absorbance was measured. Lipid peroxidation was calculated based on the molar extinction coefficient of malondialdehyde (MDA), a reliable marker of lipid peroxidation (ε = 1.56 × 10^5^/M/cm). MDA is a product of membrane phospholipids peroxidation and is generated by increased arachidonic acid metabolism. In this method one mole of MDA is condensed with two moles of TBA. The resulting coloured product is determined by spectrophotometry at λ = 535 nm following the method described by Placer et al.^31^. The level of lipid peroxidation had expressed as µM of TBARS.

### Determination of the non-enzymatic antioxidant capacity (NEAC) of blood plasma using DPPH^·^ and ABTS^·+^ radicals

Both of the using assays were based on protocols described by Bartosz and Janaszewska, with modifications^32^. The stock concentration of DPPH^**·**^ methanol solution was 500 µM. The radical stock was diluted to obtain a working reagent, with an initial absorbance of 1.2 measured at 517 nm. For the examine, 20 µL of blood plasma was diluted using 380 µL of 0.05 M phosphate-buffered saline (PBS, pH 7.4) and mixed with 400 µL of DPPH^**·**^ reagent. After 30-min. incubation at room temperature, the samples were centrifuged. The absorbance of clear supernatants was measured and compared with the blank sample (400 µL of PBS + 400 µL of DPPH^**·**^ working solution). ABTS radical reagent (2,2′-azino-bis(3-ethylbenzothiazoline-6-sulphonic acid) (10 mM) was prepared by dissolving in oxidant solution (2 mM H_2_O_2_ in 30 mM acetate buffer, pH 3.6). Next, the ABTS^**·**+^ reagent was incubated for 1 h. (room temperature, dark place) to obtain a working reagent. For the ABTS^**·**^ reduction-based analyses, in microplate wells 5 µL of blood plasma was mixed with 200 µL of 0.04 M acetate buffer (pH 5.8). Into a blank sample, 5 µL of PBS was added instead of blood plasma. The initial absorbance (A0) was immediately measured at 415 nm. Then, 20 µL of ABTS working solution was added to the wells. After 6-min incubation, the absorbance at 415 nm (A) was recorded.

The NEAC of blood plasma was calculated based on standard curves, prepared with the use of Trolox, as a reference antioxidant. Results are expressed as Radical Scavenging Activity (%) calculated as follows: RSA (%) = 100 × (A_0_ − A)/A_0_.

### Statistical analysis

The statistical analysis was performed using StatsDirect statistical software V. 2.7.2 (StatsDirect, Sale, Cheshire, UK). All values in this study were expressed as mean ± SD. The obtained results were analysed under the account of normality with Kolmogorov–Smirnov test. The homogeneity of variance in all methods was tested with the Levene test. Variances of the control and treated samples in all methods were found to be different. The significance of differences between the values was analysed for data with normal distribution by unpaired t-Student with the correction of Cochran–Cox. The significance of differences between the TaG1, T1G2 patients and controls were analysed for data with normal distribution by one-way ANOVA analysis of variance with Tukey's post-hoc test. A level of *p* < 0.05 was accepted as statistically significant.

## Results

### Oxidative/nitrative stress parameters in plasma of BC patients and healthy volunteers

**Figure 1:**
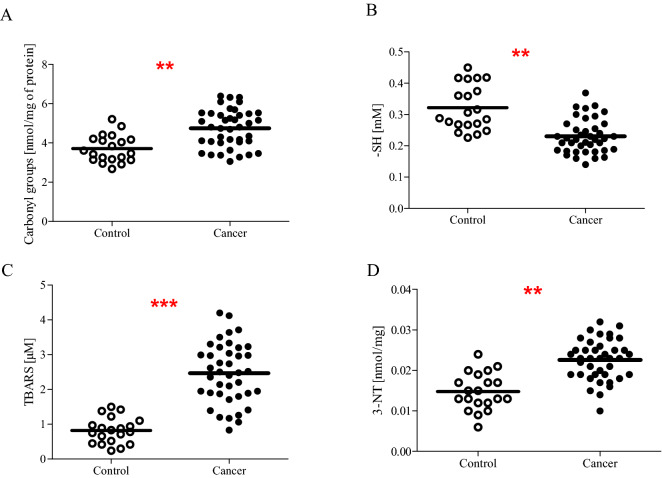
The level of carbonyl group (**A**), thiol groups (**B**), thiobarbituric acid reactive substances (**C**), 3-nitrotyrosine (**D**) in blood plasma proteins obtained from blood of cancer patients (n = 40) and control group (n = 20). Statistical analysis was performed using Student-t test with the correction of Cochran–Cox; **p < 0.01, ***p < 0.001.

In the present study, we determined plasma oxidative/nitrative stress parameters in patients with newly diagnosed bladder cancer. We noticed that all analysed biomarkers are significantly higher in enrolled patients than in healthy subjects. In our studies, we observed a statistically increased level of carbonyl groups (on average, increased by almost 30%; *p* < 0.01) (Fig. 1A), 3-NT (on average, increased beyond 50%; *p* < 0.01) (Fig. 1D) in plasma, as well as a statistically significant decreased level of protein thiol groups (on average, decreased by almost 30%; *p* < 0.001) (Fig. 1B) in plasma of BC patients in comparison to the control group. Furthermore, our comparative analysis shows that not only protein markers of oxidative/nitrative damages are significantly changed, but we also noticed an augmented level of lipid peroxidation. The average value of TBARS was threefold higher (*p* < 0.001) in plasma samples from BC patients, as compared to the control group (Fig. 1C).

**Figure 2:**
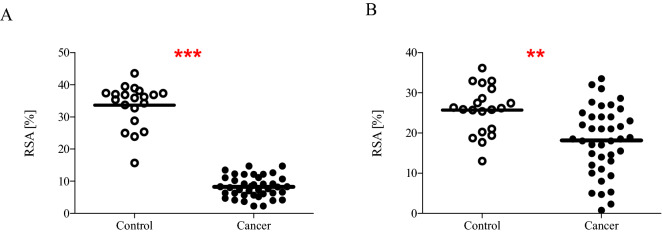
The non-enzymatic antioxidant capacity of blood plasma determined by the reduction of DPPH^**·**^ radicals (**A**) and ABTS^**·**+^ radicals (**B**) in plasma obtained from blood of cancer patients (n = 40) and control group (n = 20). Statistical analysis was performed using Student-t test with the correction of Cochran–Cox; **p < 0.01, ***p < 0.001.

Moreover, we also determined NEAC of blood plasma using DPPH^**·**^ and ABTS^**·**+^ radicals. NEAC had expressed as Radical Scavenging Activity. Figure 2A revealed that RSA was significantly lower (on average, decreased by about 75%; *p* < 0.0001) in carcinoma patients than controls. This means that the plasma of patients shows less ability to neutralize DPPH^**·**^ free radicals than the plasma of healthy volunteers. Similar results we obtained by measuring the ability of plasma to eliminate ABTS^**·**+^ radicals. The mean value of RSA was about 30% lower (*p* < 0.01) in patients than in healthy controls (Fig. 2B).

### The association of oxidative/nitrative stress parameters with the grades of the BC

Additionally, we compared the level of all measured markers of oxidative/nitrative stress in two, the most numerous groups (TaG1 and T1G2) of carcinoma patients. Data analysis according to the grades of the disease, show significant differences between TaG1 grade and T1G2 grade (Table 2). This compilation demonstrates a positive correlation between the degree of development of bladder cancer and the level of oxidative stress. The increase in the level of oxidative stress between the two groups of patients in all measurements oscillates at the level of 20–30% and is statistically significant (*p* < 0.05). The T1G2 patients were characterised by a higher level of TBARS and a lower level of protein thiol groups than TaG1 patients (*p* < 0.05). Moreover, the plasma of T1G2 patients shows less ability to neutralize DPPH^**·**^ and ABTS^**·**+^ radicals than the plasma of T1G2 patients (*p* < 0.05). Thus, with the increase in the advancement of BC grades, an increase in oxidative stress biomarkers was observed. However, in the case of 3-NT, which is mainly a marker of nitrative stress, the observed slight increase (Δ = 9%) was not statistically significant (Table 2).

**Table 2:** The level of oxidative/nitrative stress according to the grades of the disease.

	TaG1 Mean ± SD	T1G2 Mean ± SD	Δ^a^ (%)	Significance
Carbonyl groups	4.09 ± 0.92 nmol/mg	4.97 ± 0.67 nmol/mg	22	*p* < 0.05
-SH	0.273 ± 0.053 mM	0.209 ± 0.031 mM	23	*p* < 0.05
TBARS	2.08 ± 0.72 µM	2.71 ± 0.90 µM	30	*p* < 0.05
3-NT	0.021 ± 0.051 nmol/mg	0.023 ± 0.047 nmol/mg	9	*p* > 0.05
DPPH	10.76 ± 2.52%	8.23 ± 2.53%	24	*p* < 0.05
ABTS	20.68 ± 6.58%	15.01 ± 8.63%	27	*p* < 0.05

^a^The value expressed as delta represents the percentage level difference for each parameter. The delta value was calculated on the basis of the mean for each of the compared groups.

**Figure 3:**
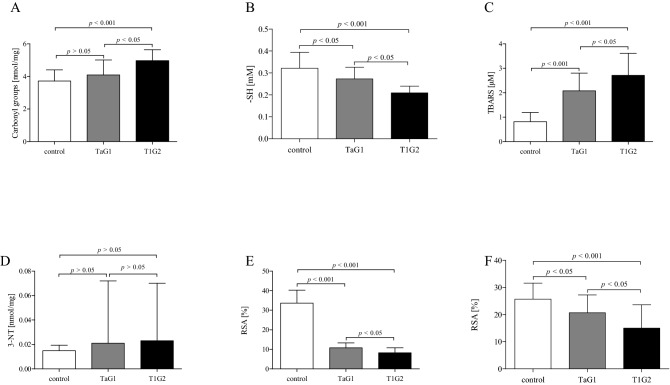
The level of carbonyl group (**A**), thiol groups (**B**), thiobarbituric acid reactive substances (**C**), 3-nitrotyrosine (**D**), the non-enzymatic antioxidant capacity determined by the reduction of DPPH^**·**^ radicals (**E**) and ABTS^**·**^ radicals (**F**) in blood plasma obtained from blood of TaG1 patients (n = 16), T1G2 patients (n = 14) and control group (n = 20). Statistical analysis was performed using one-way ANOVA analysis of variance with Tukey's post-hoc test.

Moreover, the analysis of oxidative stress marker levels in controls and patients with TaG1 and T1G2 grades showed that the most sensitive marker seems to be TBRS and non-enzymatic antioxidant capacity determined by the reduction of DPPH^**·**^ radicals (Fig. 3). For these marker levels, a more radical changes were observed for TaG1 compared to healthy volunteers. The level of TBRS about three-times increased (p < 0.001), while the mean value of RSA above four-time reduced in T1G2 patients (p < 0.001) compared to controls. However, TaG1 BC patients were characterised by nearly 1.5-times increased NEAC determined by the reduction of ABTS^**·**^ radicals, while the level of thiol groups was reduced nearly 1.5-times as compared to controls (p < 0.05).

## Discussion

The high level of ROS/RNS may be responsible for mutagenesis as well as may contribute to cancer progression and metastasis. Bladder cancer is a most heterogeneous disease, which requires independent consideration of very different stages of the tumour. It is particularly important to distinguish the low-grade Ta tumours from high-grade tumours > T1 associated with significant progression^33^.

The presented study aimed to investigate differences in the level of plasma oxidative/nitrative stress parameters in patients with bladder cancer newly diagnosed in a different stage and grade. The analysis had performed using typical oxidative stress markers, such as the plasma level of carbonyl groups, thiol groups, thiobarbituric acid reactive substances and 3-nitrotyrosine formed in blood plasma proteins, which had also compared to the control individuals. All studied markers appear early in cancer development and are relatively stable. As well, we investigated the non-enzymatic antioxidant capacity of plasma determined by the reduction of DPPH^**·**^ and ABTS^**·**+^ radicals. The levels of DPPH^**·**^ and ABTS^**·**+^ radicals reflect plasma non-enzymatic antioxidant capacity. DPPH^**·**^ radicals are used for and ABTS^**·**+^ radicals The plasma antioxidant capacity includes non-enzymatic antioxidant like vitamin A, E, C, bilirubin, albumin and reduced glutathione mainly supplied with the diet. Intensification ROS generation leads to not only to the activation and then depletion of antioxidant enzymes, but is also associated with the consumption of non-enzymatic antioxidants, supplied with the diet. We confirmed that NEAC expressed as the reduction of DPPH^**·**^ radicals (*p* < 0.001) and ABTS^**·**+^ radicals (*p* < 0.01) was decreased in plasma of BC patients. Thus, reduced NEAC, reflected by decreased reduction (neutralization) of DPPH^**·**^ and ABTS^**·**^ radicals, means that patients with BC are characterised by elevated level of ROS^34^. Moreover, many studies suggested that high dietary NEAC is inversely associated with the risk for various cancers (gastric and colorectal cancer) and cancer mortality. Therefore, an increased supply of food products with antioxidant properties may increase the efficiency of antioxidant uptake and minimize the cancer development and progression^35–37^. Patchsung et al*.* and Mazdak et al*.* also found that the urinary and blood total antioxidant status (TAS), showing an ability the plasma to defend itself against free radicals, consisting in their inactivation to substances with a neutral charge, had diminished in the BC objects relative to the controls^38,39^. Additionally, our research confirmed an approximately 25% decrease in NEAC depending on the stage of BC progression (TaG1 vs. T1G2, *p* < 0.05). The lower NEAC in patients with T1G2 than TaG1 suggests that BC progression may be associated with further intensification of oxidative processes and consumption of plasma low molecular weight antioxidants, imitated in the TaG1 stage. Cancer progression is characterising by abnormal, increased cell proliferation, which requires a lot of cell energy. The effect of high energy production in the form of ATP is excessive production of ROS. Thus, cancer progression and metastasis are associated with high ROS level. However, it should also be remembered that in very high concentrations, ROS are also toxic to cancer cells, which is using during anti-cancer therapy^40^. Moreover, our analysis suggest that the NEAC expressed as the reduction of DPPH^**·**^ radicals seems to be one of the most sensitive biomarkers of oxidative stress, which is reduced fourfold in patients with TaG1 stage as compared to healthy volunteers (p < 0.001). This may suggest that in the development of BC, low molecular weight antioxidants supplied with the diet are the first to use up the most, which are the first line of defence against free radicals.

In bladder epithelium cells, the lipids, including membrane phospholipids, and proteins are the main target of ROS and RNS action^7^. Thus, previous studies confirmed that patients with BC were characterised by an increased generation of the lipid peroxidation products, including malondialdehyde (MDA) in serum and 8-iso-prostaglandin F2 α (8-iso-PGF2 α) in urine as compared to the control group^41,42^. MDA is a product of membrane phospholipid peroxidation which is the result of the elevated cyclooxygenase-2 (COX-2) expression^43^. Interestingly, *COX-2* expression was detected in only neoplastic tissue but no normal urothelial cell, which additionally emphasizes the close correlation between a high harmful level of ROS and BC development^43^. Moreover, *COX-2* mRNA level was inversely correlated with the occurrence of recurrence of non-muscle invasive bladder cancer and associated with advancing grade and T stage of superficial transitional cell bladder carcinoma^43–45^. However, no correlation was observed between the level of an oxidation product of arachidonic acid, 8-iso-PGF2 α, and the degree of BC malignancy and invasiveness^42^. This difference between MDA and 8-iso-PGF2 α may indicate that MDA can be used as a potential diagnostic and prognostic marker in BC. In the case of cancerous bladder tissues, it has been observed an increased level of thiobarbituric acid reactive substances (TBARS)^46,47^. TBARS are the by-product of lipid peroxidation, and the TBARS assay allows measurement of MDA presented in the sample. Thus, the increased TBARS level corresponds to the increased MDA concentration and reflects the level of lipid peroxidation. Similarly, our findings indicated a significantly was threefold higher level of TBARS (*p* < 0.001) in plasma of BC patients than in the control group. Moreover, the presented results proved that plasma TBARS level was positive correlated with the BC development degree (*p* < 0.05). Lepara and colleagues (2020) also found that, the patients from T1 and T2–T4 groups were characterised by higher the serum MDA level than in the patients from Ta group. Moreover, a positive correlation was confirmed between tumour size and serum MDA level in patients with bladder cancer^41^. Thus, the intensification of pro-oxidative processes of lipids, especially membrane phospholipids, may be associated with the degree of malignancy and invasiveness of BC. Consequently, oxidative damage to membrane lipids may lead to the granulocyte adhesion to the endothelium and then xanthine–oxidase activation. This activation causes increased hydrogen peroxide production in a vicious circle^48^. A high ROS level may also induce NF-κB (nuclear factor kappa-light-chain-enhancer of activated B cells) which may modulate transcription of proinflammatory cytokines, including interleukins 6 and 8 (*IL-6* and *IL-8*)^49,50^. Interestingly, *IL-8* expression may be correlated with the metastatic potential of human transitional cell carcinoma^50^. Additionally, NF-κB activation causes the transcription induction of anti-apoptotic and proangiogenic factors, such as B-cell lymphoma-extra large (Bcl-xL) and matrix metalloproteinases 9 (MMP-9). Bcl-xL induction prevents the release of mitochondrial contents including cytochrome c, which in effect prevents activation of caspases and finally, apoptosis. In turn, MMP-9 is involved in the extracellular matrix degradation, and thus plays a crucial role in the angiogenesis and tumour progression^18,51^. Moreover, a high ROS level may also enhance the binding of Sp1/Sp3 (transcription factors) to the *VEGF-A* (vascular endothelial growth factor A) promoter, which may be a crucial mechanism of transactivates the *VEGF-A* induced expression. A high VEGF-A expression impacts increased vascular permeability, induces angiogenesis, vasculogenesis and endothelial cell growth, promotes cell migration, and inhibits apoptosis^52^. Thus, oxidative stress may be a factor that initiates the activation of other pathways involved in neoplastic transformation, including inflammation, apoptosis and angiogenesis. Moreover, our results suggest that, besides NEAC expressed as the reduction of DPPH^•^ radicals, TBARS is also a highly sensitive marker of oxidative stress, which is elevated threefold in patients with TaG1 stage as compared to healthy volunteers (p < 0.001). This may suggest that in the development of BC the most critical to oxidative damage are lipids, especially membrane phospholipids, which are exposed to both external and intra-cellular exposure.

In addition to lipid peroxidation, protein damages have observed in the course of cancer. The oxidative and nitrative failures of proteins cause the peptide backbone cleavage, cross-linking and/or modifications of the side chain of every amino acid^53^. Moreover, the great number of protein damages are irreparable and may lead to disorders of protein structure, which impact its function, including inhibition of enzymatic activities, a misfolding, the increased ability of proteins to aggregation and proteolysis, and altered immunogenicity^54^. Thus, in the presented study, oxidative damage of proteins demonstrated as protein carbonylation, 3-nitrotyrosine formation, reduced level of thiol residues have been investigated. The main carbonyl products of metal-catalysed protein oxidation are glutamic semialdehyde, a product of arginine oxidation, aminoadipic semialdehyde, a product of lysine oxidation, 2-pyrrolidine, a product of histidine oxidation and 2-amino-3-ketobutyric acid, a product of oxidation of threonine. The transformation of amino acids into carbonyl derivatives gives rise to the loss of enzymatic activity^55,56^. Proteins exposed to ROS-induced carbonylation include membrane proteins, electron transport chain proteins, and endoplasmic reticulum proteins. The carbonylation protein of complex I and III subunits is associated with the loss of their catalytic activity, which in turn may lead to further generation of ROS by mitochondria. In turn, tubulin carbonylation leads to microtubule disassembly and instability, which can be used in the development of new anticancer therapies^57–60^. The carbonylation may also cause thioredoxin inactivation, but not lead to ASK1 (apoptosis signal-regulating kinase 1) release. The carbonylated thioredoxin remains associated with the active site of the N-terminus of ASK1, thus inhibiting the kinase and blocking the induction of the apoptosis process dependent on ASK1, thus leading to cancer progression^61^. Our study confirmed that BC patients showed an increased level of plasma carbonyl groups to the control (*p* < 0.01). These findings are consistent with the previous study indicating the increased levels of plasma and serum protein carbonyls in BC patients as compared to the control^36,62^. Moreover, our study has also shown that the level of oxidative damage to plasma proteins expressed as carbonylation is significantly associated with the degree of BC progression (TaG1 vs. T1G2; p < 0.05). Perhaps this is a consequence of the permanent inactivation of ASK1, thus enabling the blockade of tumour cell apoptosis and the induction of BC progression.

We also proved the dependence of protein oxidative damage degree on BC progression by measuring the content of thiol residues in plasma. The protein cysteine thiols of most eukaryotic cells are characterised by the greatest sensitivity to fluctuations in the cellular redox state^26^. The thiol-disulphide balance plays a crucial role in antioxidant defence, detoxification, apoptosis, regulation of enzymatic activity and intracellular signalling mechanisms^63^. Under physiological conditions, the cytosol has a highly reducing environment character that maintains thiol residues in the reduced state. In turn, ROS and RNS could lead to the initial formation of sulfenic acids, which then could be converted into disulphides^26^. Thus, our results confirmed that the level of thiol residues was also lower in BC group than in healthy controls (*p* < 0.01). Our findings are consistent with the report of Yilmaz et al., who detected the level of total thiol groups and protein-bound thiol groups were lower in patients with the BC than controls. Interestingly, the level of protein-bound thiol groups in patients with invasive bladder cancer revealed a more decline than in the non-invasive group^62^. We also confirmed that the level of thiol residues was negatively correlated with the invasive degree of bladder cancer (*p* < 0.05). Importantly, our study included a wider group of patients—both women and men, while the changes described by Yilmaz were limited only to the male population. One of the key thiol-containing proteins is glutathione, which is considered as one of the most important cellular "thiol buffer", and its level is lower in the course of the various cancer^64,65^. The decreased level of reduced glutathione may be crucial for the processes of cancer invasion associated with the type IV collagenase pathway. Type IV collagenase plays a crucial role in this tumour cell-mediated extracellular matrix proteolysis. In turn, glutathione is involved in the repression of this enzyme and inhibits tumour invasion. Thus obtained results suggest that an increased protein thiol oxidation causes an expenditure of glutathione reserves and abolishes the repression of type IV collagenase. This is followed by the tumour progression to surrounding tissues, as the glutathione reservoir in the tumour and the consumable reduced thiol groups decrease. Therefore, a reduced level of thiol groups in patients with invasive tumour may be used as a prognostic factor^66^.

The BC development may also be associated with increased NO level observed in BC tissue, urine and serum, which may be correlated with an increased expression of *iNOS* in bladder tumoral tissue. Interestingly, this overexpression was associated with the transition to more advanced stages of BC^67,68^. The hyperproduction of NO with increased ROS generation, especially superoxide (O_2_^**·**−^), leads to the formulation of ONOO^−^, a highly reactive compound that causes nitration of tyrosine residues and leads to 3-nitrotyrosine (3-NT) production^24,69^. 3-NT is the early detectable biomarker found in patients suffering from cancers, such as prostate cancer^70^. Our study showed that the plasma level of 3-NT was increased in patients with BC as compared to controls (*p* < 0.01). Soini et al*.* found that 36% of the BC cases showed 3-NT positivity^71^. Moreover, patients with tumours characterising by high 3-NT expression had a worse survival in among bladder tumour patients. However, we found no correlation between the 3-NT level and the grades of the disease. Similarly, Wolf and colleagues found no relation between nitric oxide synthase expression and bladder cancer severity or stage^72^. The differences between previous results might be attributed to the dual role of nitric oxide during epithelial proliferation and cancer progression in the bladder. Previous studies showed that the accelerated cell proliferation was caused by endogenous NO in the bladder epithelium and BC cells while the repression of cell proliferation was caused by inducible NO stimulated by cytokines^72,73^. NO plays a variety of roles in carcinogenesis, including is involved in DNA damage, oncogenes activation, regulation of apoptosis and metastasis, inhibition of DNA repair enzymes and inhibit of tumour suppressors. However, NO also portrays anti-tumorigenic effects by utilizing the immune defences mechanisms in cancers^74–76^.

Concluding, the described above ROS overproduction manifested in increased plasma levels of protein carbonyls, 3-nitrotyrosine, lipid peroxidation as well as reduced thiol group concentration, may lead to cancer transformation of cells. Increased ROS level can be involved in cell proliferation, apoptosis, and tumorigenesis by modulating the expression of transcription factors, enzymes, and structural proteins as well as DNA damage, including DNA strand breakage, DNA–DNA crosslink, or DNA–protein crosslink. Moreover, ROS can also involve in BC progression by inducing redox-sensitive pro-tumorigenic and pro-metastatic genes such as *VEGF* and *MMP-9*^16^. Thus, very high ROS concentration might be positively correlated with an advanced tumour stage and grade.

## Conclusion

The level of a potential biomarker, including carbonyl groups, thiol groups and were correlated with the BC invasive stage, the most sensitive turned out to be thiobarbituric acid reactive substances and the non-enzymatic antioxidant capacity determined by the reduction of DPPH^**·**^ radicals. Due to the high sensitivity and specificity of the proposed biomarkers, they may facilitate early and accurate diagnosis of BC. However, the presented study should be conducted in a larger cohort of patients with BC. This may enable the estimation of the prognostic values for the examined parameters, and thus allow their practical application in BC diagnostics.
